# Transient prenatal ruxolitinib treatment suppresses astrogenesis during development and improves learning and memory in adult mice

**DOI:** 10.1038/s41598-021-83222-z

**Published:** 2021-02-15

**Authors:** Han-Chung Lee, Hamizun Hamzah, Melody Pui-Yee Leong, Hadri Md Yusof, Omar Habib, Shahidee Zainal Abidin, Eryse Amira Seth, Siong-Meng Lim, Sharmili Vidyadaran, Mohamad Aris Mohd Moklas, Maizaton Atmadini Abdullah, Norshariza Nordin, Zurina Hassan, Pike-See Cheah, King-Hwa Ling

**Affiliations:** 1 Department of Biomedical Sciences, Faculty of Medicine and Health Sciences, Universiti Putra Malaysia, 43400 UPM Serdang, Selangor Malaysia; 2 Department of Biotechnology, Faculty of Science, Technology, Engineering and Mathematics, International University of Malaya-Wales, 50480 Kuala Lumpur, Malaysia; 3 Faculty of Science and Marine Environment, Universiti Malaysia Terengganu, 21030 Kuala Nerus, Terengganu Malaysia; 4 Department of Human Anatomy, Faculty of Medicine and Health Sciences, Universiti Putra Malaysia, 43400 UPM Serdang, Selangor Malaysia; 5 Collaborative Drug Discovery Research, Faculty of Pharmacy, Universiti Teknologi MARA, Cawangan Selangor, Kampus Puncak Alam, 42300 Bandar Puncak Alam, Selangor Malaysia; 6 Department of Pathology, Faculty of Medicine and Health Sciences, Universiti Putra Malaysia, 43400 UPM Serdang, Selangor Malaysia; 7 Institute of Biosciences, Universiti Putra Malaysia, 43400 UPM Serdang, Selangor Malaysia; 8 Genetics and Regenerative Medicine Research Centre, Faculty of Medicine and Health Sciences, Universiti Putra Malaysia, 43400 UPM Serdang, Selangor Malaysia; 9 Centre for Drug Research, Universiti Sains Malaysia, 11800 Penang, Malaysia

**Keywords:** Model vertebrates, Mouse, Drug development

## Abstract

Ruxolitinib is the first janus kinase 1 (JAK1) and JAK2 inhibitor that was approved by the United States Food and Drug Administration (FDA) agency for the treatment of myeloproliferative neoplasms. The drug targets the JAK/STAT signalling pathway, which is critical in regulating the gliogenesis process during nervous system development. In the study, we assessed the effect of non-maternal toxic dosages of ruxolitinib (0–30 mg/kg/day between E7.5-E20.5) on the brain of the developing mouse embryos. While the pregnant mice did not show any apparent adverse effects, the Gfap protein marker for glial cells and *S100β* mRNA marker for astrocytes were reduced in the postnatal day (P) 1.5 pups' brains. Gfap expression and Gfap^+^ cells were also suppressed in the differentiating neurospheres culture treated with ruxolitinib. Compared to the control group, adult mice treated with ruxolitinib prenatally showed no changes in motor coordination, locomotor function, and recognition memory. However, increased explorative behaviour within an open field and improved spatial learning and long-term memory retention were observed in the treated group. We demonstrated transplacental effects of ruxolitinib on astrogenesis, suggesting the potential use of ruxolitinib to revert pathological conditions caused by gliogenic-shift in early brain development such as Down and Noonan syndromes.

## Introduction

Ruxolitinib (initially called INCB18424) is the first janus kinase 1 (JAK1) and JAK2 inhibitor that was approved by the United States Food and Drug Administration (FDA) agency for the treatment of myelofibrosis^1^ and polycythaemia vera^2^. It has been shown beneficial to patients with intermediate-2 or high-risk myelofibrosis^3^. In recent years, ruxolitinib prescription has been expanded to various diseases due to its immunosuppressive and anti-inflammatory effects. Ruxolitinib has been effective against steroid-refractory graft-versus-host disease^4^ and coccidioidomycosis^5^. During the coronavirus disease 2019 (COVID-19) pandemic, ruxolitinib was used to treat severe COVID-19 cases in a single-blind, randomised controlled trial involving multi centres^6^.

Ruxolitinib, a type I inhibitor, exerts its inhibitory effect by competing with adenosine triphosphate (ATP) for the ATP-binding catalytic site of the JAK kinase domain. JAK comprises of four tyrosine kinases (JAK1, JAK2, JAK3 and tyrosine kinase (TYK) 2) and the signal transducer and activator of transcription (STAT) factors containing a family of seven members (STAT1, STAT2, STAT3, STAT4, STAT5A, STAT5B and STAT6)^7^. The cell surface receptors with associated JAKs are brought into proximity when the ligands bind to the receptor allowing the JAKs to phosphorylate each other. The trans-phosphorylation lead to the activation of JAKs, which phosphorylates the receptor and creates a binding site for SH2 domains of the STATs^8,9^. When STATs bind to the receptor, the conserved tyrosine residue near the C-terminus is phosphorylated and lead to STATs dimerisation through the conserved SH2 domain. The phosphorylated STAT dimers translocate into the nucleus and bind to the promoter of the target gene to initiate transcriptional activity^10^. Several studies showed that ruxolitinib treated cells, such as pancreatic tumour cells derived from p53-null mice, resulted in p-JAK2 and p-STAT3 suppression^11^. Other studies also showed that the expression level of p-STAT3 in human non-small cell lung carcinoma cells was reduced when they were treated with ruxolitinib^12^.

The JAK-STAT signalling pathway is critical for several cellular processes such as cell proliferation, differentiation and apoptosis^13^. This pathway is also involved in the regulation of the development of haematopoietic cells and immune responses^14^. Notably, the JAK and STAT proteins are highly expressed in the developing brain^15^, implicating their important role in brain development. The JAK-STAT signalling pathway is essential in regulating the fate of neural progenitor cells (NPCs), whereby it promotes NPCs differentiation into glial lineage^16^. During gliogenesis, the STAT3 transcription factor is activated through phosphorylation by JAKs and competitively binds to the p300/CBP coactivator proteins leading to Notch-mediated activation of gliogenic fate that produce astrocytes^17^. Dysfunctional astrocytes due to the impairment of astrocytogenesis have been implicated in various disorders related to the central nervous system (CNS) such as Alzheimer's disease, epilepsy, brain tumour, major depression and Down syndrome (DS)^10,18^. Interestingly, a neurogenic-to-gliogenic shift during neurodevelopment has been shown in Down^17^ and Noonan syndromes^19^ characterised by increased astrogenesis or preferential NPCs differentiation into astrocytes^19,20^. Increased astrocytes were observed in the hippocampus^21^ and frontal lobe of human DS foetuses^22^. The loss of neurons to astrocytes ratio in early neurodevelopment has been postulated as one of the reasons leading to intellectual disabilities and impaired cognitive functions. Taken together, targeting the JAK-STAT signalling pathway using ruxolitinib could regulate astrogenesis to restore the neurons-astrocytes balance.

Ruxolitinib is a pregnancy category C teratogen. Although there is no evidence of teratogenicity, in animal studies, pregnant rats given this medication have shown approximately 9% reduction in foetal weight at the highest and maternally toxic dose of 60 mg/kg/day^23^. Currently, there are no adequate and well-controlled studies with ruxolitinib in pregnant women^2^. In addition, Haile and colleagues^24^ recently demonstrated that ruxolitinib was able to cross the blood–brain barrier (BBB) and reduced astrogliosis in the brains of mice with HIV encephalitis. The BBB development starts in as early as E7.5 in mice or gestational day (GD) 17–21 in human during neurulation. The functional BBB develops in a spatiotemporal manner and peaks shortly after birth in rodents or gestational weeks 35 in human^25,26^. Taken together, ruxolitinib, when used at a lower or non-maternal toxic dose (< 60 mg/kg/day) could effectively target JAK/STAT signalling pathway in early developing embryonic/foetal nervous system, whereby the BBB remains underdeveloped. The drug could be beneficial to revert pathological conditions seen in Down or Noonan syndromes that affect the early brain development or at time when BBB is compromised to alleviate gliosis aggression.

In this study, we assessed the effect of non-maternal toxic doses of ruxolitinib (up to 30 mg/kg/day) on the central nervous system of the developing mouse embryos. The transient treatment was aimed to suppress or delay astrogenesis in the developing mouse brain as a proof of concept study to revert neurogenic-to-gliogenic shift seen in Down or Noonan syndromes. Our study demonstrated the transplacental effect of ruxolitinib, which led to the reduction of the astrocytic marker in early postnatal pups and a marked improvement in spatial learning and memory retention in early adulthood a potential use of ruxolitinib to target JAK/STAT signalling pathway in early brain development.

## Materials and methods

### Ethical clearance, compliance, animal husbandry and handling

All mice described in this study were handled, and all procedures, experiments and tissue procurements were performed in accordance with guidelines and regulations approved by the Institutional Animal Care and Use Committee (IACUC) of Universiti Putra Malaysia (UPM) under reference number UPM/IACUC/AUP-R086/2015. The study was carried out in compliance with the Animal Research: Reporting of In Vivo Experiments (ARRIVE) guidelines.

All mice were housed in an individually ventilated cage with unidirectional sterile airflow at constant temperatures (25 °C) with a 12:12 h light–dark cycle in the mouse room facility, Genetics and Regenerative Medicine Research Centre (GRMRC), UPM. The mice had access to standard animal feed (Altromin 1324) and clean water ad libitum. During treatment, each animal was restrained either by using a cappy cone or by firmly grasping the scruff of its neck for proper immobilisation.

### Ruxolitinib preparation and dosages

For the animal study, ruxolitinib (MedChem Express LLC, USA) was dissolved in dimethyl sulfoxide (DMSO) at 60 mg/ml concentration. The stock was kept at -20 °C and only thawed to room temperature before use. The drug was then vortexed and mixed with 1% (w/v) methylcellulose (Amresco) prepared in saline, a more palatable aqueous vehicle, before oral gavage. The transient treatment of ruxolitinib for mice was defined by the non-maternally toxic doses previously published in the FDA report^23^. The maximum dose of ruxolitinib used in the study was 30 mg/kg of body weight. For ex vivo neurospheres study, ruxolitinib (Invivogen) stock solution (20 mM) was prepared in DMSO. Working solution of ruxolitinib (100 µM) was prepared in 0.5% DMSO prior to all treatment. A range of ruxolitinib concentrations (0–10 µM) was used by adding the appropriate amount of the working solution into the culture media.

### Prenatal ruxolitinib treatment and brain protein lysate collection

Six groups of C57BL/6 aged postnatal day (P) 56–70 female mice (n = 5 per group) were set up for overnight mating with stud males. Vaginal plugs were checked the next morning for successful mating and termed gestational or embryonic day (E) 0.5. Between gestation period at E7.5 and E20.5, a control group (G1) was orally fed once daily with 1% (w/v) methylcellulose in saline as vehicle using oral gavage. Whilst, five other groups were fed daily at different doses of ruxolitinib in methylcellulose: 1 mg/kg (G2), 5 mg/kg (G3), 10 mg/kg (G4), 15 mg/kg (G5) and 30 mg/kg (G6) body weight. Oral or intragastric gavage was performed by using a 22G steel gavage with ball tip (Harvard Apparatus). After birth, P1.5 pups were weighed, anaesthetised on ice-cold petri dish and decapitated. The P1.5 brain was harvested in cold PBS, weighed and flash-frozen on dry ice for protein lysate preparation.

### Toxicity analysis of ruxolitinib in pregnant mice

Bodyweight, feed, and water consumptions of the pregnant mice were recorded at E1.5 and measured at ten-day intervals before delivery. At post-delivery P1.5, the mothers were euthanised using inhalation of 4% (v/v) isoflurane, followed by cervical dislocation. The whole blood was collected through intracardiac technique into a 1.5 ml centrifuge tubes containing 1% EDTA to prevent clotting. The blood samples were sent to Veterinary Laboratory Service Unit (VLSU) at the Faculty of Veterinary Medicine, UPM for liver and kidney function biochemical analyses such as the levels of alanine aminotransferase (ALT), aspartate aminotransferase (AST), total protein, albumin, bilirubin, blood urea, sodium, potassium, phosphorus and chloride. Multiple vital organs such as liver, kidney and spleen were dissected for haematoxylin and eosin staining followed by a histopathological assessment by a pathologist.

### Ex vivo neurosphere culture from embryonic cortical tissue

E15.5 embryos were collected from time-mated C57BL/6 mice. The left and right hemispheres of the cerebral cortex were harvested from the mouse embryos. After removal of meninges and visible blood vessels from the cortex, the dissected tissue was dissociated into single cells in 0.05% trypsin solution (Gibco) by gentle resuspension. Subsequently, cells were centrifuged for 5 min at 1,000 rpm, re-suspended, and plated in a medium consisting of Neurobasal Medium (Gibco), 1X B-27 Supplement (Gibco), 20 ng/mL epidermal growth factor (EGF, Gibco), 20 ng/mL fibroblast growth factor (FGF, Gibco), 1% GlutaMAX (Gibco), 2 mM L-Glutamine (200 mM, Gibco) and 1% penicillin/streptomycin (10,000 U/ml, Gibco). Neurospheres were sub-cultured when the size measured between 100 µm – 150 µm in diameter.

### Differentiation of neurospheres

A 6-well plate was coated with 50 ug/ml of poly-l-lysine (Sigma) in phosphate-buffered saline (PBS) for 1 h at 37 °C.The solution was discarded and the plate washed twice with PBS before air-dried. Subsequently, the poly-L-lysine coated wells were overlaid with 10ug/ml of laminin (Sigma) in PBS and incubated at 37 °C for 1 h before washed with PBS twice. The wells were seeded with 5 × 10^5^ dissociated cells and were differentiated for 7 days in vitro (DIV) in NSC differentiation medium with treatment. The treatment consisted of DMSO only (vehicle control group) or ruxolitinib mixture (6 groups between 0.1 µM and 10 µM concentrations) in Neurobasal Medium (Gibco) supplemented with 1X B-27 Supplement (Gibco), 1% foetal bovine serum (FBS, Gibco), 1% GlutaMAX (Gibco), 2 mM L-Glutamine (200 mM, Gibco) and 1% penicillin/streptomycin (10,000U/ml, Gibco).

### Protein lysates preparation

Protein lysate was extracted from the frozen whole-brain sample with 250 µl of ice-cold complete radioimmunoprecipitation (RIPA) lysis buffer (Merck Millipore) at 5X tissue volume. The complete RIPA buffer consisted of 1X protease inhibitor (Calbiochem) and 1X phosphatase inhibitor (Roche Life Science) to prevent and minimise protein degradation. Brain sample was homogenised on ice by pipetting up and down followed by occasional vigorous vortexes over a 40 min incubation on ice. Subsequently, the lysate was centrifuged at 16,000 g for 30 min. The supernatant was collected and for immunoblotting assay.

To collect protein lysate from the differentiating neurosphere, the media was removed after 7 DIV, and the cell was washed with PBS. The cells were lysed by resuspension in 200 µl ice-cold complete RIPA lysis buffer and incubated for 30 min on ice. The lysate was centrifuged at 16,000 g for 20 min before the supernatant was collected for immunoblotting assay.

### Immunoblotting assay

Lowry protein assay (Bio-Rad Laboratories) was used to quantitate protein concentrations according to the manufacturer's protocol. A total of 20 µg protein sample cocktail in Laemmli buffer (Bio-Rad Laboratories) and beta-mercaptoethanol (Bio-Rad Laboratories) were loaded into Biorad Mini-PROTEAN TGX precast gels (Bio-Rad Laboratories) for electrophoresis using the Mini-PROTEAN Tetra cells system (Bio-Rad Laboratories). Following electrophoresis, gels were placed into Bio-Rad Transfer Pack (Bio-Rad Laboratories), and proteins were transferred onto a nitrocellulose membrane using Trans-Blot Turbo Transfer System for seven minutes using midi transfer pack at 2.5 A, up to 25 V. Blots were then blocked with 5% (w/v) bovine serum albumin (BSA) in 1X tris buffered saline with 0.1% (v/v) Tween 20 (TBST) for 1 h followed by three washes in 1X TBST at 15 min each on a see-saw shaker in room temperature. The blot was then incubated with primary antibody at 4 °C, on a shaker overnight based on the dilution factor stated in Table 1. On the next day, the blot was washed three times with 1X TBST for 15 min each at room temperature before incubated with secondary antibody for 1 h at room temperature. Specific signals on blots were then detected with Advansta WesternBright Sirius electrochemiluminescence (ECL) Western blotting detection kit for 5 min before image capturing by using the Syngene Gbox Chemi HR1-4 gel documentation system (Syngene). Pixels of the migrated bands were quantified using ImageJ software (https://imagej.nih.gov/).

**Table 1:** List of antibodies for western blot or immunocytochemistry.

Antibody	Host	Target MW (kDa)	Dilution factor	Application	Manufacturer #Cat. ID
Anti-JAK1	Rabbit polyclonal	~ 130	1:500	WB1	Cell Signaling #3332
Anti-JAK2	Rabbit polyclonal	~ 125	1:500 1:5000	WB1 WB2	Merck Millipore #06–255
Anti-Phospho-JAK1 (Tyr1034/1035)	Rabbit polyclonal	~ 130	1:1000	WB1	Cell Signaling #3331
Anti-Phospho-JAK2 (Tyr1007/1008)	Rabbit polyclonal	~ 130	1:1000 1:5000	WB1 WB2	Merck Millipore #09–275
Anti-Gfap	Mouse monoclonal	~ 50	1:1000 1:500	WB1 WB2; ICC	Merck Millipore #MAB360
Anti-Tuj1	Rabbit polyclonal	~ 55	1:1000 1:500	WB1 WB2; ICC	Sigma-Aldrich #T2200
Anti-β-actin	Rabbit polyclonal	~ 45	1:1000	WB2	Cell Signaling #4967
Anti-β-actin	Goat polyclonal	~ 45	1:1000	WB1	Santa Cruz #sc-1616
Anti-Gapdh	Mouse monoclonal	~ 36	1:1000	WB1	Abcam #ab125247
Anti-rabbit IgG, HRP-linked	Goat	-	1:2500	WB	Cell Signaling #7074
Anti-mouse IgG, HRP-linked	Goat	-	1:3000	WB	Santa Cruz #sc-2005
Anti-goat IgG, HRP-linked	Donkey	-	1:3000	WB	Santa Cruz #sc-2020
Alexa Fluor 488 donkey anti-rabbit IgG	Donkey	-	1:1000	ICC	Life technologies #A21206
Alexa Fluor 555 donkey anti-mouse	Donkey	-	1:1000	ICC	Life Technologies #A31570

WB1 refers to immunoblotting for brain tissue lysates from P1.5 pups (Fig. 2); WB2 refers to immunoblotting for protein lysates from differentiating neurospheres (Fig. 3); ICC refers to immunocytochemistry.

### Immunocytochemistry staining

The differentiating neurospheres were fixed using 4% paraformaldehyde (PFA) in PBS, pH7.0 for 20 min at room temperature. After fixation, the cells were washed three times with PBS. To improve antibody penetration, the cells were permeabilised with 0.2% (v/v) Triton X-100 diluted in PBS for 10 min and followed by three washes in PBS for 5 min. Afterwards, cells were blocked in 3% (w/v) BSA with 0.2% (v/v) Triton X-100 in PBS for 1 h at room temperature followed by an overnight incubation at 4 °C with primary antibodies in 1% (w/v) BSA in PBST (Table 1). Subsequently, the cells were washed three times in PBS. The primary antibodies were detected using the fluorescently labelled secondary antibodies diluted in 1% (w/v) BSA for 1 h at room temperature in a dark chamber followed by three washes in PBS in the dark. The cells were then incubated with 0.2 μg/mL 4′,6-diamidino-2-phenylindole (DAPI) in PBS for 20 min at room temperature for nuclei counterstaining. Background controls were carried out using samples labelled with secondary antibodies only. Stained cells were visualised under the Olympus BX51 fluorescent microscope (Olympus).

### Total RNA preparation, cDNA synthesis and quantitative polymerase chain reaction (qPCR)

Total RNA was extracted from flash-frozen P1.5 whole brain tissue by using TRIzol reagent (Invitrogen) according to the manufacturer's protocol. A total of 3 µg of total RNA was reverse transcribed into cDNA using oligo d[T]_20_ using a SuperScript IV Reverse Transcriptase kit (Invitrogen) according to the manufacturer's instructions. Quantitative PCR analysis on *Tuj1* (*Tubb3*), *NeuN* (*Rbfox3*), *Gfap*, *S100β*, *Olig1* and *Olig2* gene expression was performed based on a method described previously^27^.

### Behavioural assays

#### Mice and experimental setup

Pups that were either treated with methylcellulose or 30 mg/kg/day ruxolitinib (between E7.5 to E21) were housed without further interventions until they reached the age of early adulthood (P60-P80) before subjecting them to a battery of behavioural assays. The treated and untreated mice were generated from 3–5 different experiment sets and consisted of 16 mice per group. All behavioural tests were performed between 0700 and 1900 h during the light cycle under normal light conditions (60–100 lx). In all the apparatuses, faecal materials and urine trails were cleaned with 70% ethanol and allowed to dry in between each behavioural test. Prior to euthanisation, animals were subjected to a visual test. Data of mice with visual impairment were excluded from all data analyses.

All mice were subjected to the following tests in chronology:

- Day 1–3: Grip strength & rotarod
- Day 4: Rest day
- Day 5–6: Hanging wire test
- Day 8: Open field test
- Day 9: Novel object recognition test (2 identical objects)
- Day 10: Novel object recognition test (1 original and 1 novel objects)
- Day 11–13: Rest day
- Day 14–18: Morris water maze (training)
- Day 19: Rest day
- Day 20: Morris water maze (probe trial) & visual test

#### Grip strength, hanging wire and rotarod tests

Forelimb grip strength was assessed using BIOSEB automated grip meter (In Vivo Research Instruments) according to the method previously described^28^. The mice were suspended by the holding on the tail so that their forelimb could reach out for metal mesh. At a horizontal plane, the mice were gently pulled away by their tails until their grips were broken. The maximum force (N) exerted by the grips right at the breaking point was recorded. A total of three different readings were recorded in three different days to allow sufficient time for strength recovery after each trial.

In addition to the grip test, the mice were also subjected to the hanging wire test. The hanging wire test aimed to evaluate the strength of the forelimb muscles. The "falls and reaches method" was adopted according to a method described previously^29^. A hanging apparatus was designed consisting of two vertical stands (35 cm above the ground) and insulated metal wire (smooth wire) (length 30 cm, 0.2 cm in diameter) suspended between the two stands. A rectangular plastic cage (15 cm × 17 cm × 30 cm) filled with soft bedding was placed underneath the hanging wire to absorb the impact of the fall. The mice were handled by the tail and brought near to the midpoint of the wire and allowed to be suspended by the forelimbs only. As soon as the mice were suspended correctly, the timer was started. The number of falls within 180 s were recorded. The experiment was performed two times over 2 days.

To assess for motor coordination and balance, the mice were placed on a commercially available rotarod apparatus (Ugo Basile). The motor coordination was assessed using both fixed (4 rpm for 60 s) and accelerated (4–64 rpm over 120 s) velocities. Whenever the mice fell off the treadmill or clung to the rotating rod, the trial would be halted, and the elapsed time was recorded. The experiment was performed three times over 3 days.

#### Locomotor function test

The explorative and the level of anxiety of the mice were assessed in an open field. The open field was made of a box measuring 50 cm × 30 cm × 30 cm with opaque walls. The base of the field was divided into ten clearly marked areas (numbered as Area 1 to10). Areas 1–4 and 6–9 formed the outer ring of the field and were considered as the "safe" areas for the mice because they were adjacent to the walls of the box. It is the natural aversion to brightly lit open area of the mice to avoid the inner/centre court of the field (areas 5 and 10). Mice were placed in the middle of the field (area 10), and their locomotor activities in the field were recorded. The time and distance travelled in each area throughout a 10 min course were analysed by SMART Video Tracking System (Harvard Apparatus).

#### Object recognition test

Using the same box for open field test, the mice were assessed for object recognition based on the affinity of mice for novelty according to a method described previously^30^. On day 1, the mice were exposed to 2 familiar objects (an opaque yellow cap plastic specimen jar, known A) placed at the left (A-L) and right (A-R) side of the field. The mice were placed at a spot with equal distance from both objects and were allowed to explore for up to 3 min in the field. The exploration of objects A-L and A-R were counted. On day 2 or the test day, the mice were placed into the same field, but with the object A-L replaced with object B-L (a novel, plastic toy with a pumpkin shape of similar base diameter and height as object A). The mice were allowed to explore in the field for up to 3 min, and the exploration counts of both novel (B-L) and familiar (A-R) objects were recorded. Exploration of an object was counted when the mice touched or sniffed the object within 2 cm from the object. Mice that explored both objects less than a total of 7 counts were removed from data analysis. The recognition index (RI) or the absolute difference of exploration counts between the different objects in day 2 was calculated by subtracting the counts in object B-L (novel object) with object A-R (familiar object). In addition, the discriminatory ratio (DR) or the relative difference of exploration counts between the different objects was determined by dividing the RI with the total counts for both objects B-L and A-R. A DR above zero indicates animals exploring the novel object more than the familiar object.

#### Morris water maze test

The mice were subjected to the Morris water maze test for spatial learning and memory assessment. The water maze was made of a black round tank with a diameter of 120 cm and depth 60 cm. Four main directions were marked on the pool: north, west, south and east. Inside the pool, a large cue was printed in black on a white A4 paper and laminated for waterproofing. A " " cue was placed at north, whereas " ", " " and " " cues were placed at east, south and west, respectively. The escape platform (10 cm × 10 cm) was placed at the middle of the north-east quadrant and was 1 cm submerged underwater. White tempera paint was added into the water to make it opaque so it could conceal the escape platform. The temperature of the water was maintained at around 21–23 °C, which was lower than the room temperature to invoke the mice to search for the escape platform. The same experimenter wearing a white laboratory coat was assigned to handle all the mice throughout the training and testing phases. After the mouse was placed in the pool, the experimenter stood behind a curtain for the rest of the session.

Mice were trained for five consecutive days with 2–3 trials per day during the training phase. Each mouse was trained 14 times over the 5-day course from different entry points. In each training, the mouse was placed in the pool with its nose facing away from the experimenter near the wall of the entry point. The mouse was given 1 min to find the platform and stay on it for 5 s. If the mouse failed to find the platform at the end of the 1 min, the experimenter would guide the mouse (from the nosepiece direction) to the platform and then allow the mouse to stay on the platform for 1 min to observe additional cues around it. After the training session, the mouse was returned to its prewarmed cage for at least 3 min. All floating excreta were removed from the pool/tank before next experiment.

During the test phase (probe trial), the escape platform was removed from the pool. The time of crossing the former platform quadrant and the total time of crossing all quadrants were recorded for 1 min. Both training and probe trial phases were recorded as a video and subsequently analysed by SMART Video Tracking System (Harvard Apparatus).

#### Visual test using the Morris water maze

After testing all the mice, a visual test was performed to make sure that the mice were free from visual impairment. For this test, the escape platform was returned to its original location (middle of the north-east quadrant) and made visible. Each mouse was tested from two different entry points (west and south) with 1 min per test. Mice that failed to reach the escape platform from both entry points within the given time were considered visually impaired and excluded from all behavioural analysis.

### Data analysis

All statistical analyses and graph illustration were carried out using Prism 7.0 for Windows (GraphPad). In general, parametric tests were used to test 2 (t-test) or more datasets (ANOVA) whereby no significant differences were found in their variances. In the case where the standard deviations or variances were significantly different (F test) between datasets, nonparametric analyses were performed in replacement of t-test (Mann–Whitney test to compare the ranks) or ANOVA (Kruskal–Wallis test). Detailed statistical comparisons were elaborated in the caption of each figure. In all the analysis, a corrected *p*-value < 0.05 was considered as statistically significant.

## Results

### Ruxolitinib does not cause any apparent toxicity towards the pregnant mice

**Figure 1:**
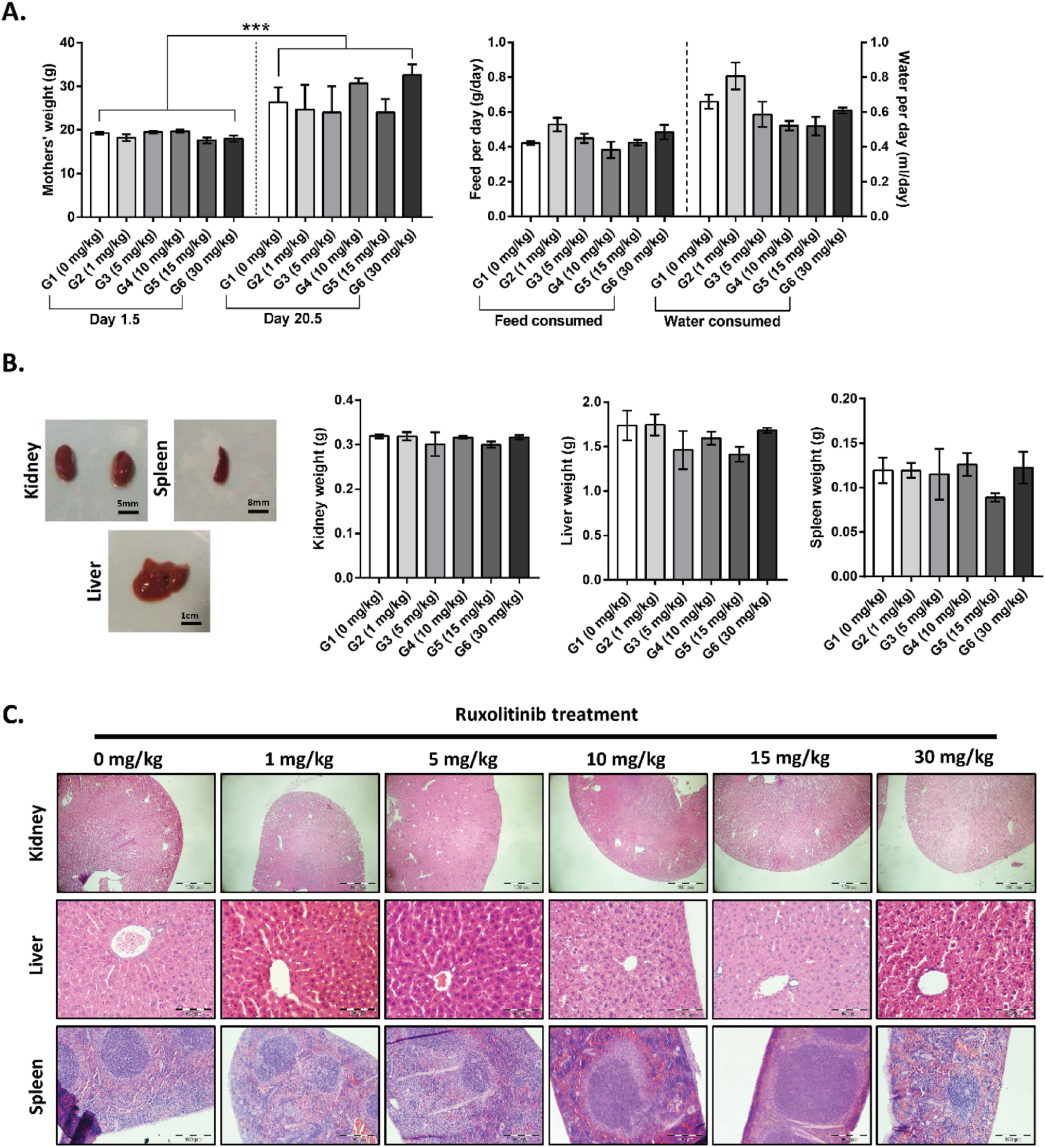
Behavioural, gross anatomical and histopathological assessments of pregnant mice underwent transient ruxolitinib treatment. (**A**) Analysis of weight (n = 3–7 per group for 1.5 day post-coitum and n = 3–6 per group for 20.5 day post-coitum; One-way ANOVA with Sidak's multiple comparisons), and normalised feed and water consumptions (n = 3–6 per group; One-way ANOVA with Dunnett's multiple comparisons) between G1 (0 mg/kg) vehicle control group and treated groups. (**B**) Representative figure for gross anatomical assessment and the weight of the kidney, liver and spleen (n = 3 per group; One-way ANOVA with Dunnett's multiple comparisons) harvested from G1-G6 pregnant mice. (**C**) Representative haematoxylin and eosin staining of kidney, liver, and spleen sections (n = 3 per group) from G1-G6 pregnant mice. All values represent mean ± SEM; *** denotes adjusted *p* < 0.001.

We first established the safety of ruxolitinib (≤ 30 mg/kg) as a transient treatment to suppress gliogenesis in the developing embryos. The potential toxicity towards the pregnant mice in behavioural, physiological, and histopathological changes was evaluated. We assessed the weight changes, feed, and water consumption of the pregnant mice as the indications of potential psychological stress due to daily oral gavage (from day 7.5 to 20.5 post-mating). On 1.5 days post-mating, the female mice's starting body weight did not differ significantly (17.6–19.7 g, n = 3–7 per group) among groups. On 20.5 days post-mating, all the pregnant mice gained weight without significant differences among the group (24.0–32.7 g, n = 3–6 per group, One-way ANOVA with Sidak's multiple comparisons). However, the weight gained between day 1.5 and 20.5 post-mating was expectedly and significantly different (Fig. 1A), reflecting a healthy increase of body weight during the gestational period (One-way ANOVA with Sidak's multiple comparisons). No differences were noted in the normalised feed and water consumptions in all the treated groups compared to control (G1) (Fig. 1A, One-way ANOVA with Dunnett's multiple comparisons). The findings suggest that the daily oral treatment of ruxolitinib had no significant effect on mice behavioural changes.

Clinical biochemistry analysis of mothers' plasma samples for liver and renal profiles relay interesting findings. The liver profile was generally normal, with all the readings from the control group (G1) within the physiological reference intervals (Table 2, n = 3 per group, One-way ANOVA with Dunnett's multiple comparisons). Although the profiles of ALT, AST and albumin of some of the treated groups correlated with the increasing dosage of the ruxolitinib fed, these values were not statistically significant. On the contrary, the levels of bilirubin were higher in all treated groups when compared to control (G1, 0.73 ± 0.09 mg/dl) with a significant increase (~ eightfold, adjusted *p* = 0.0064) at 5 mg/kg (G3, 5.77 ± 0.56 mg/dl) and 10 mg/kg (G4, 5.77 ± 1.01 mg/dl) of ruxolitinib treatment but not at higher dosages. However, the renal function profiles indicated higher potassium, sodium, and phosphate levels in the controls (G1). Surprisingly, in all the treated groups, both potassium (G2-G6, 4.53–5.70 mmol/l vs G1, 23.27 ± 1.26 mmol/l) and phosphate (G2-G6, 1.55–1.90 mmol/l vs G1, 3.63 ± 0.28 mmol/l) were significantly reduced (adjusted *p* < 0.001) to a level almost comparable to the reference intervals. In contrast, sodium was seen increased significantly only in 30 mg/kg (G6, 217.00 ± 16.65 mmol/l vs G1, 180.70 ± 4.91 mmol/l) ruxolitinib-treated group (adjusted *p* = 0.0468). Other renal parameters such as chloride, urea and creatinine were not significantly different from the control mice.

**Table 2:** Biochemical blood analysis.

Blood profiles (*reference range*)^^^^	G1 (0 mg/kg)	G2 (1 mg/kg)	G3 (5 mg/kg)	G4 (10 mg/kg)	G5 (15 mg/kg)	G6 (30 mg/kg)
Alanine aminotransferase (ALT) (*22–32 U/l*)	*31.33* ± *1.76*	*26.00* ± *1.53*	35.33 ± 7.80	53.33 ± 25.10	76.00 ± 54.78	39.67 ± 8.21
Aspartate aminotransferase (AST) (*40–60 U/l*)	*59.20* ± *4.25*	67.97 ± 19.33	75.67 ± 7.68	33.33 ± 13.08	80.87 ± 69.97	81.30 ± 30.50
Total protein (*47.5–52.0 g/l*)	*48.43* ± *1.73*	*50.83* ± *1.19*	*48.37* ± *0.32*	*48.13* ± *2.35*	53.53 ± 2.58	47.20 ± 2.52
Bilirubin (*0.17–0.83 mg/dl*)^@^	*0.73* ± *0.09*	1.87 ± 1.52	5.77** ± 0.56	5.77** ± 1.01	2.27 ± 0.37	3.30 ± 0.91
Albumin (*26–29 g/L*)	*29.30* ± *0.95*	*28.93* ± *0.32*	*27.60* ± *1.71*	31.17 ± 2.26	35.37 ± 1.85	33.33 ± 1.24
Potassium (*3.5–4.1 mmol/l*)	23.27 ± 1.26	5.70*** ± 0.10	4.53*** ± 0.58	5.30*** ± 0.00	5.10*** ± 0.80	4.80*** ± 0.50
Sodium (*143–148 mmol/l*)	180.70 ± 4.91	182.30 ± 4.70	189.70 ± 7.86	193.30 ± 5.24	191.30 ± 6.77	217.00* ± 16.65
Chloride (*107–111 mmol/l*)	*107.70* ± *1.76*	104.00 ± 0.58	104.30 ± 2.73	98.00 ± 2.52	95.67* ± 3.76	99.00 ± 3.51
Phosphate (*1.2–1.8 mmol/L*)	3.63 ± 0.28	1.90*** ± 0.06	1.70*** ± *0.17*	1.90*** ± 0.10	1.55*** ± *0.05*	1.57*** ± *0.12*
Blood Urea (*9.27–12.09 mmol/l*)	6.57 ± 0.20	7.30 ± 0.93	6.93 ± 0.82	*9.23* ± *1.04*	*10.33* ± *1.91*	8.87 ± 1.22
Creatinine (*26.5–35.4 μmol/l*)^#^	45.00 ± 10.26	17.33 ± 2.33	*30.00* ± *2.31*	46.33 ± 24.92	65.67 ± 34.17	*32.67* ± *3.53*

^^^^ these reference intervals for clinical biochemistry profiles were obtained from P90-P135 (n = 1486) C57BL/6 and C3HeB/FeJ female mice^31^, with exception to the profile labelled with ^@^, which were obtained from 8-, 26- and 52-week old (n = 4, 7 and 8 per age group, respectively) C57BL/6 female mice^32^, whereas the profile labelled with ^#^ were obtained from inbred (n = 10) and outbred (n = 30) 4-week old female mice^33^. Italic readings are within the reference intervals. All values represent in mean ± SEM.

* Denotes adjusted *p* < 0.05, ** denotes adjusted *p* < 0.01 and *** denotes adjusted *p* < 0.001 when compared to G1 (0 mg/kg) group (n = 3 per group, based on One-way ANOVA with Dunnett's multiple comparisons).

The kidney, liver, and spleen were harvested for gross anatomical and histopathological assessments to complement biochemical blood profiles' findings. There were no significant changes to the size, weight, appearance, or discolouration of these organs in all the groups (Fig. 1B, based on One-way ANOVA with Dunnett's multiple comparisons). At 400X magnification, haematoxylin and eosin-stained kidney, liver and spleen sections indicated normal renal glomeruli and tubular system, hepatocytes, and splenic parenchyma (red and white pulps) in all groups. There was no observable immune cells infiltration in all the tissue sections, indicating that these organs were not under acute or chronic inflammation process (Fig. 1C). Taken together, the findings based on behavioural, biochemical and histopathological assessments indicate that ruxolitinib transient treatment is safe when prescribed orally throughout the pregnancy in mice.

### Transient ruxolitinib treatment suppressed Gfap marker in the P1.5 brain and differentiating neurospheres ex vivo

**Figure 2:**
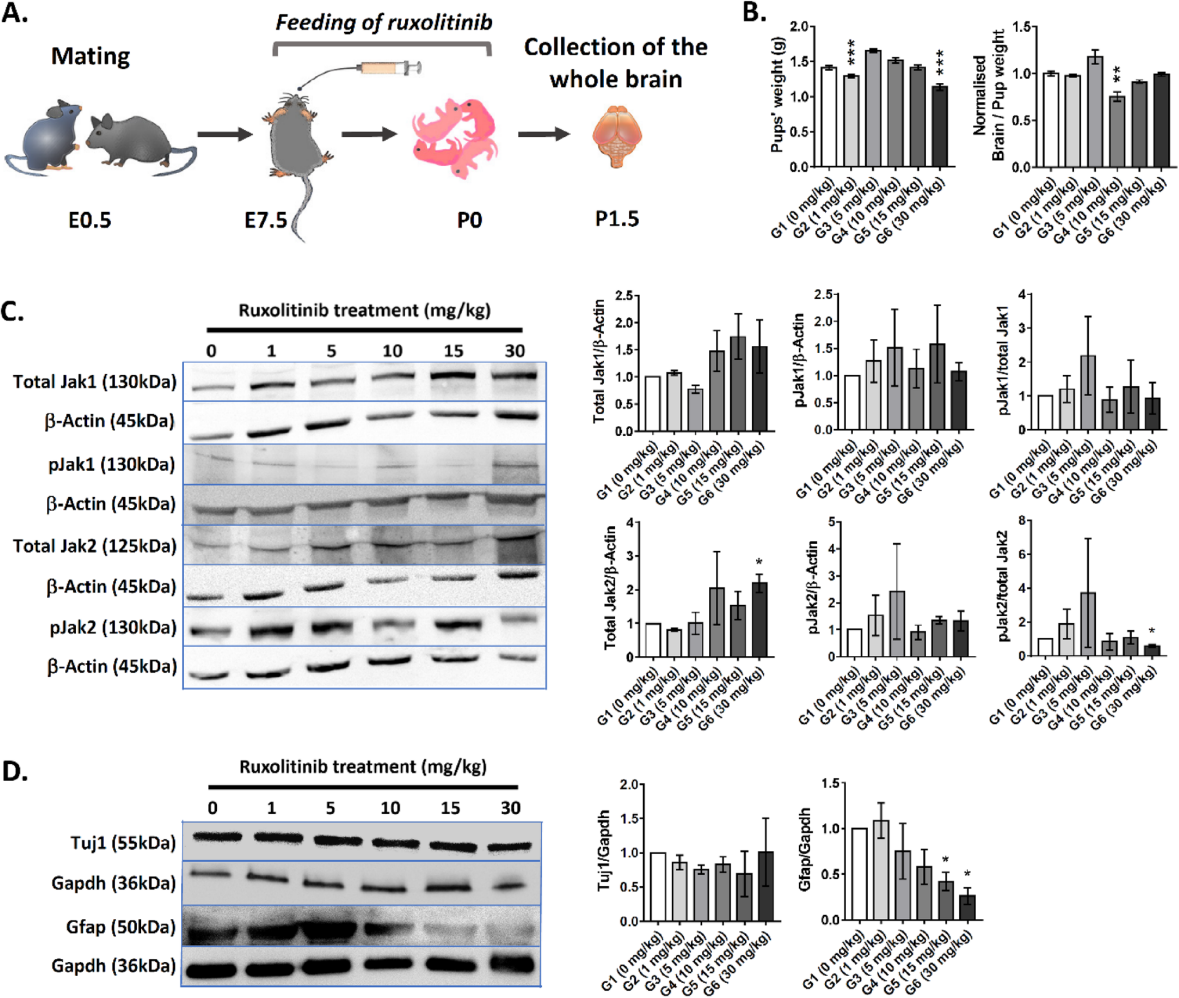
Assessment of gliogenic-suppression effect of ruxolitinib in postnatal brain. (**A**) A schematic diagram depicting the study outline for prenatal transient ruxolitinib treatment. (**B**) The weight measurement of P1.5 pups (n = 6–21 per group; One-way ANOVA with Dunnett's multiple comparisons) and their brain (n = 4–21 per group; Kruskal–Wallis test with Dunn's multiple comparisons) in vehicle control (G1) and all the treated groups (G2-G6). (**C**) Immunoblotting analysis for total Jak1 and Jak2, and phosphorylated Jak1 and Jak2 expression in all groups. The corresponding relative pixelation analysis of immunoblotting bands is shown right to the blots (n = 3 per group; unpaired, two-tailed Welsh's t test). (**D**) Immunoblotting analysis for Tuj1 and Gfap markers in P1.5 whole brains in all groups and the corresponding relative pixelation analysis of immunoblotting bands is shown right to the blots (n = 3 per group; unpaired, two-tailed Welsh's t test). All values represent mean ± SEM; * denotes adjusted *p* < 0.05; ** denotes adjusted *p* < 0.01; *** denotes adjusted *p* < 0.001. See Figs. S1 and S2 for original immunoblots (Supplementary information).

To evaluate the effect of prenatal transient ruxolitinib treatment on gliogenesis in P1.5 pups, mated female mice were administered orally with ruxolitinib between E7.5 and E20.5 gestational period (Fig. 2A). In the past, ruxolitinib treatment at 60 mg/kg in rats showed no obvious pathological phenotypes but gave birth to pups with reduced weight. In our transient treatment, we observed that the pups from G2 (1 mg/kg, n = 8, 1.296 ± 0.023 g) and G6 (30 mg/kg, n = 6, 1.139 ± 0.048 g) treated groups exhibited significantly reduced weight (-10.2% and -19.4%, respectively) when compared to controls (G1, n = 21, 1.413 ± 0.031 g, adjusted *p* < 0.001, One-way ANOVA with Dunnett's multiple comparisons) (Fig. 2B). However, the harvested P1.5 pups' brains were found to be significantly smaller in G4 (10 mg/kg, n = 4, 0.755 ± 0.047 g) with normalised weight ~ 25% lower than controls (G1, n = 21, 1.000 ± 0.025 g, adjusted *p* = 0.0063, Kruskal–Wallis test with Dunn's multiple comparisons). The random occasions of reduced pups' and the brain weight indicate a possible differential metabolism towards ruxolitinib.

Ruxolitinib has been shown to alter the levels of pJak1 or pJak2 and their function in Jak-Stat signalling activation. We assessed the level of total Jaks and their corresponding Tyr1007/1008 phosphorylation status. Generally, the total Jak1, total Jak2, pJak1 and pJak2 (Fig. 2C) in the P1.5 whole brain did not differ significantly in all treated groups than controls except G6 (30 mg/kg). All comparisons were based on two-tailed unpaired t-test with Welsh's correction (n = 3 per group). The level of total Jak2 was upregulated by ~ 2.2-fold (adjusted *p* = 0.0481). Although the corresponding pJak2 was not different from control, the ratio of pJak2/total Jak2 was ~ 42% reduced in G6 (30 mg/kg, adjusted *p* = 0.0480) group. The findings suggest a possible inhibition of Tyr1007/1008 phosphorylation by ruxolitinib despite the surge of total Jak2 level that could be compensatory. We further assessed whether ruxolitinib treatment suppresses the Jak-Stat signalling-mediated astrogenesis in developing brains. We evaluated the levels of Tuj1 (a marker for postmitotic neurons) and Gfap (a marker for astroglial cells) expression in P1.5 pups' brains (Fig. 2D). All comparisons were based on two-tailed unpaired t-test with Welsh's correction (n = 3 per group). Tuj1 expression levels were unaltered in all the groups. In contrast, Gfap levels were significantly reduced in G5 (15 mg/kg, ~ 2.4-fold, adjusted *p* = 0.0279) and G6 (30 mg/kg, ~ 3.8-fold, adjusted *p* = 0.0142) ruxolitinib-treated groups. Assessment of mRNA expression levels indicated a significant increase in *Tuj1* (*Tubb3*, ~ 2.95-fold, adjusted *p* = 0.0290) and *NeuN* (*Rbfox3*, ~ 4.3-fold, adjusted *p* = 0.0473) neuronal markers in G6 treated-group when compared to the control. On the other hand, a significant decrease in *S100β* astrocyte marker was observed in G5 (~ 3.0-fold, adjusted *p* = 0.0340) and G6 (~ 3.9-fold, adjusted *p* = 0.0183) treated-groups when compared control. *Gfap* was seen with a downward trend and no differences in the levels of *Olig1* and *Olig2* oligodendrocyte markers as ruxolitinib concentration increases (Supplementary Information, Fig. S3). The finding suggests that the transient ruxolitinib treatment via the mothers could delay astrogenesis in the embryos based on both protein and mRNA expression levels.

**Figure 3:**
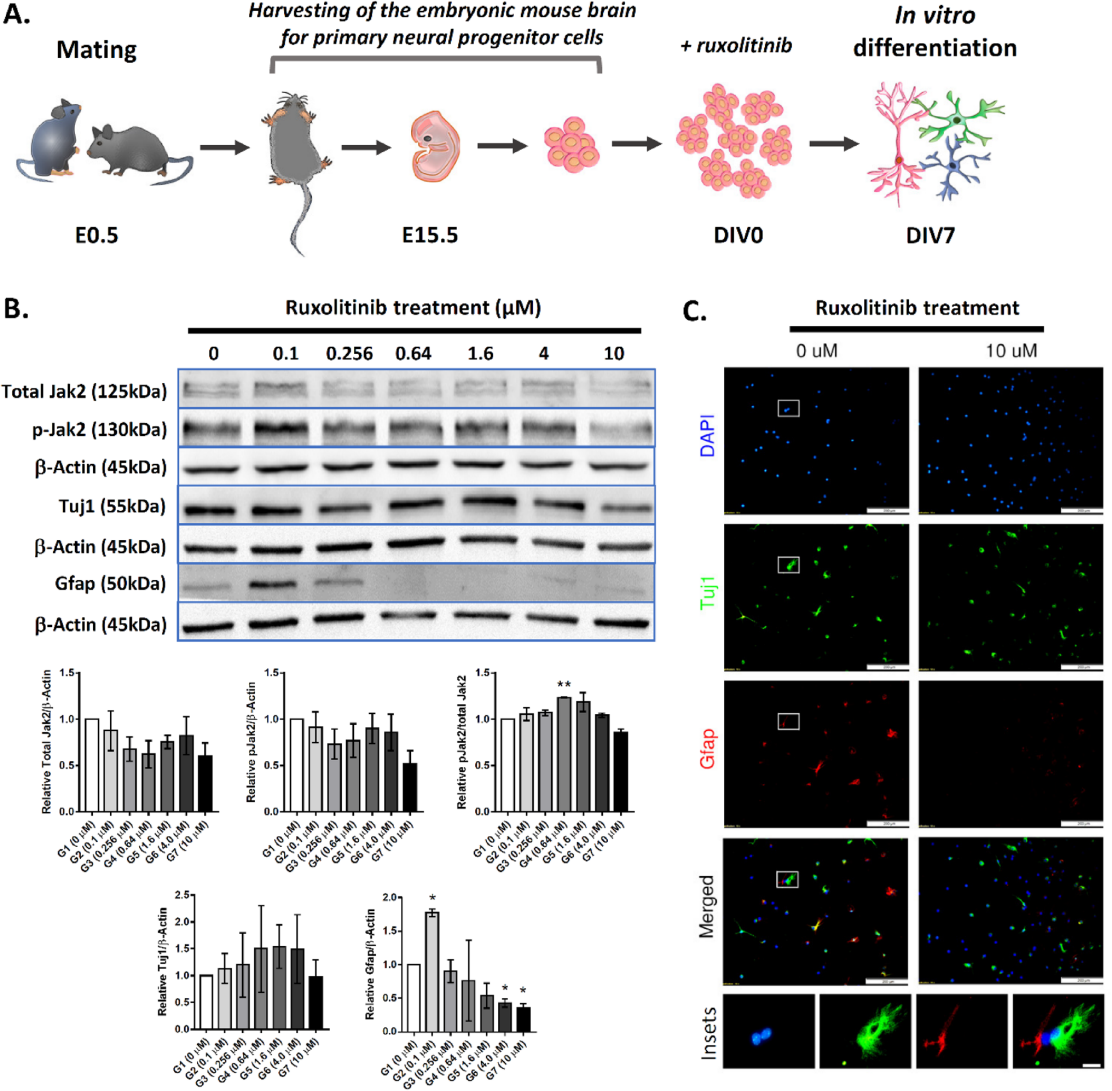
Assessment of the gliogenic-suppression effect of ruxolitinib in differentiating neurospheres ex vivo. (**A**) A schematic diagram depicting the study outline of ex vivo differentiation of neurospheres for ruxolitinib treatment. (**B**) Immunoblotting analysis for total Jak2, phosphorylated Jak2 (n = 2 per group), Tuj1 and Gfap (n = 2 per group) in vehicle control (G1) and all treated groups (G2-G7). The corresponding pixelation analysis of immunoblotting bands is shown below to the blots (n = 2 per group; Welsh's t-test). (**C**) Immunocytochemistry staining of Tuj1 and Gfap in differentiating neurospheres in control and 10 μM ruxolitinib treated groups (n = 2 per group). The higher magnification of insets in the control group is shown at the bottom of the micrographs. The colour of the insets corresponds to the DAPI (blue), Tuj1 (green) or Gfap (red). Scale bars represent 200 μm in all micrographs whereas 20 μm in the insets. All values represent mean ± SEM; * denotes adjusted *p* < 0.05; ** denotes adjusted *p* < 0.01. See Fig. S4 for original immunoblots (Supplementary information).

To recapitulate the findings seen in P1.5 pups' brains in a better-defined cellular system, we harvested neural progenitor cells from the E15.5 developing cortex and cultured them ex vivo as neurospheres (Fig. 3A). The neurospheres were then neurodifferentiated up to 7 days with and without ruxolitinib treatment. Immunoblotting analysis for total Jak2 and pJak2 indicated no differences between all the treated groups with the control (Fig. 3B) (n = 2 per group, one-tailed, unpaired t-test with Welsh's correction). Validating the findings from the P1.5 brains, the corresponding Gfap markers in differentiating neurospheres were reduced significantly in G6 (4 μM, ~ 2.4-fold, adjusted *p* = 0.0330) and G7 (10 μM, ~ 2.8-fold, adjusted *p* = 0.0315) ruxolitinib-treated groups. Similar to the P1.5 whole-brain lysate analysis, Tuj1 did not differ significantly in all groups ex vivo. Immunocytochemistry assessment of the differentiating cells after 7-day of ruxolitinib treatment indicate very low to no presence of Gfap^+^ cells, whereas Tuj1^+^ cells remained similar in both control and treated groups (Fig. 3C). In summary, both in vivo and ex vivo experiments indicated a consistent suppression of astrogenesis by ruxolitinib. However, the regulation of Jak-Stat signalling pathway by ruxolitinib remains hard to interpret and beyond a simplistic regulation of Jak1 and Jak2 phosphorylation.

### Improved spatial learning and memory in adult mice subjected to prenatal transient ruxolitinib treatment

**Figure 4:**
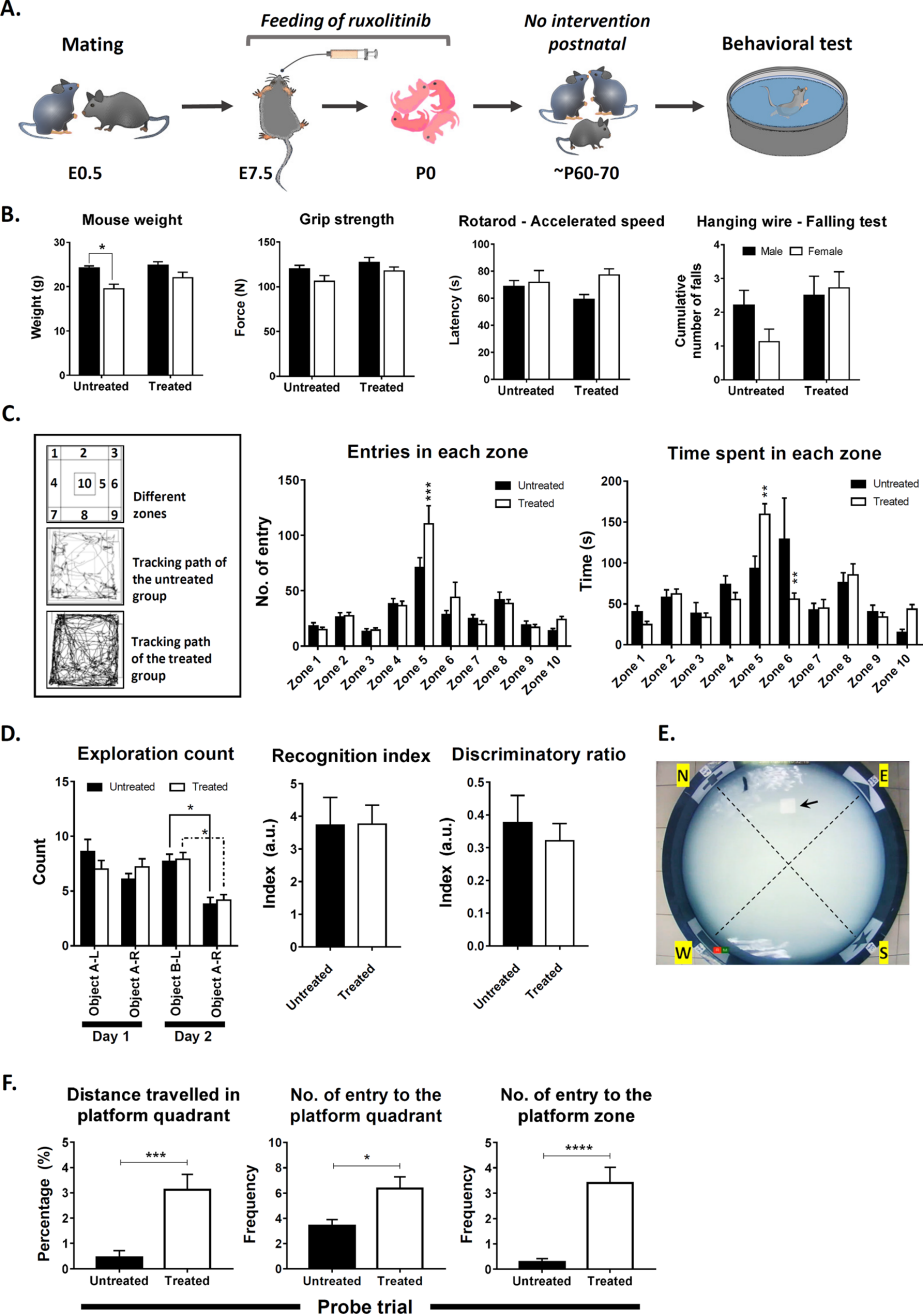
Behavioral assessments on young adult mice underwent prenatal transient ruxolitinib treatment. (**A**) A schematic diagram depicting the prenatal transient ruxolitinib treatment from E7.5 to E20.5 gestational period. After delivery, the pups were allowed to grow and mature into young adulthood without further intervention before behavioural assessments. (**B**) The measurements of mouse weight, grip strength, forelimb strength (hanging wire test) and locomotor coordination (rotarod). All comparisons (Control, n = 14; treated group, n = 16) were tested with Two-way ANOVA with Sidak's multiple comparisons**. **(**C**) Open field test for explorative behaviour and the general level of hyperactivity/anxiety. Representative mouse locomotion tracking for both the vehicle control and treated groups. The number of entry and time spent in each zone (zones 1–10) within the open field are presented to the right. All comparisons (Control, n = 14; treated group, n = 16) were tested with Two-way ANOVA with Sidak's multiple comparisons. (**D**) Novel object recognition assessment for the mouse capability to recognise a familiar and discriminate a novel object. Mice with < 7 total exploration count were removed from the analysis leaving n = 13 for control and n = 15 for the treated group on day 1 whereas on day 2, n = 10 for control and n = 12 for the treated group. The number of times the vehicle control and treated groups explore each object on day 1 (A-L and A-R) and day 2 (B-L and A-R) was tested with Two-way ANOVA with Tukey's multiple comparisons. The recognition and discriminatory indices for both control and treated groups are depicted on the right and tested with two-tailed, unpaired t-test with Welsh's correction. (**E**) The bird view of the Morris water maze setup. The arrow indicates the hidden escape platform in the middle of the north-east quadrant. (**F**) The proportion of distance travelled to the north-east quadrant, number of entry to the north-east quadrant and the escape platform zone during the probe trial on day 7. N = 14 for the vehicle control group and n = 16 for the treated group in the probe trial session were compared using two-tailed, exact Mann Whitney test. All values represent mean ± SEM; * denotes adjusted *p* < 0.05; ** denotes adjusted *p* < 0.01; *** denotes adjusted *p* < 0.001; **** denotes adjusted *p* < 0.0001.

We evaluated the effect of early gliogenesis suppression by transient ruxolitinib treatment on various behavioural parameters when the pups reached young adulthood. The mated female mice were given 30 mg/kg of ruxolitinib between E7.5 and E20.5 gestational period. After delivery of the pups, the treated mothers were allowed to nurse and wean the pups. After weaning, the mice were housed under standard conditions until they reached P60-P80 of age for a battery of behavioural tests (Fig. 4A). Of the 16 mice per group, 2 mice were found visually impaired in control and removed from all behavioural data analysis. Hence, 10 males and 4 females in the control group, whereas 7 males and 9 females in the treated group. All parameters were tested with Two-way ANOVA with Sidak's multiple comparisons. The mice's weight right before the beginning of any behavioural test was significantly lower in the female (-19.4%, adjusted *p* = 0.0277) than the male in the control group. No gender weight differences were observed in the treated group. Similarly, we observed no significant differences between gender in the same group or differences between control and treated groups in grip strength, the latency of falls via rotarod test and the cumulative fall in the hanging wire test (Fig. 4B). The findings suggest that the prenatal transient ruxolitinib treatment did not affect the overall muscle strength and locomotor performance.

We subjected the mice to an open field test with 10 pre-marked zones to assess their general explorative behaviour and hyperactivity/anxiety level based on the time and distance travelled in these zones (Fig. 4C). Both groups travelled similar distance in the field (control = 83.23 ± 8.95 m vs treated = 85.6 ± 9.63 m), but the treated group showed a significant increase in the number of entry (~ 1.55-fold, adjusted *p* = 0.0002), time spent (+ 1.70-fold, adjusted *p* = 0.0097) and proportion of distance travelled (+ 1.46-fold, adjusted *p* = 0.0009) to zone 5 of the field. On the contrary, mice from the treated group showed a significant decrease in time spent (~ 2.30-fold, adjusted *p* = 0.0026) to zone 6 within the field. Zone 5 and 10 are more open area than zones 1–4 and 6–9, which bordering the container walls serving as safer zones for natural mouse navigation. Although the transient ruxolitinib treatment did not affect the mice's general locomotor activity, the treated mice seemed less anxious and willing to explore a more open zone than the control mice.

Next, we assessed the recognition memory or commonly termed the mice's short-term memory capability to recognise familiar objects and discriminate a novel object through the novel object recognition test (Fig. 4D). Mice with < 7 total exploration count were removed from the analysis leaving n = 13 for control and n = 15 for the treated group on day 1 whereas on day 2, n = 10 for control and n = 12 for the treated group. Both control and treated groups habituated well with two identical objects on day 1 of the experiment. Mice from both groups did not show any differences in exploration count towards identical objects, A-L and A-R throughout a 10 min training session on day 1. On day 2, after a 24 h retention interval, both control (~ 2.0-fold, adjusted *p* = 0.0263) and treated (~ 1.9-fold, adjusted *p* = 0.0154) mice showed a significant increase in exploration count towards the novel object, B-L according to Two-way ANOVA with Tukey's multiple comparisons test. However, the recognition index and the discriminatory ratio of both groups did not differ from each other. The observations suggested that prenatal transient ruxolitinib treatment did not alter or affect the intermediate retention of recognition memory.

Finally, the treatment's effect on spatial learning and long-term memory of the treated mice was assessed using the Morris water maze test (Fig. 4E). The escape latency of the treated mice was significantly lower than the control group indicating an increase in spatial learning capability when compared to the control mice. Ruxolitinib treatment accounted for ~ 23.7% of the total variance between the groups (*p* < 0.0001, Two-way ANOVA with Sidak's multiple comparisons) (Supplementary information, Fig. S5). The escaped latency in the treated group becomes prominent from the third day of training and onwards. Overall, the escaped latency curve indicate minimal (~ 10%) learning among control mice. This was due to the inaccurate baseline of escaped latency which was capped at 60 s on the first trial during the training. After the training, the mice were rested for 48 h before being tested for their long-term memory retention. During the probe trial, the pool's total distance travelled was comparable (adjusted *p* = 0.2000 based on two-tailed, unpaired t-test with Welsh's correction) between the control (18.27 ± 2.149 m) and treated (22.01 ± 1.865 m) groups. The treated group, however, showed a significant increase in the proportion of distance travelled (~ 7.0-fold, *p* = 0.0002 based on two-tailed, exact Mann–Whitney test) and the number of entry (~ 1.9-fold, *p* = 0.0271) to the north-east quadrant (platform quadrant) (Fig. 4F). The number of entries to the platform zone was more prominent in the treated group with ~ 12-fold more than the control group (*p* < 0.0001). However, the Tuj1 and Gfap expression in these mice's hippocampus did not differ between the control and treated groups (data not shown). The increased exploration of the treated group within the escaped platform zone and the north-east quadrant indicated that the prenatal transient ruxolitinib treatment led to improved spatial learning and long-term memory retention.

## Discussion

USFDA approved ruxolitinib to treat myelofibrosis^1^ and then polycythaemia vera^2^, both are haematopoetic malignancies. General toxicology assessment of ruxolitinib in a single-dose (100 mg/kg) in rats indicated that the drug was well tolerated with no observations of acute toxicity whereas the repeat-dose (5–60 mg/kg/day) up to 6 months led to reduced body weight (-12%), levels of circulating leukocytes including lymphoid depletion in multiple lymph nodes and spleen, which changes were generally reversible^23^. In our study, however, we neither observed any changes in term of the mouse body nor the organs' weights when the animals were treated with 30 mg/kg/day of ruxolitinib. In addition, no changes in mouse behaviour during gestation and histopathological aggression towards the kidney, liver, and spleen. The observations were most likely attributed to the lower dosage used over a shorter period, the transient treatment approach that we aimed to achieve in the study.

Toxicology screening of ruxolitinib treatment on the clinical biochemistry profiles has been lacking. In our study, liver function profiles were elevated in all treated mice, but none of the protein levels was statistically significant except bilirubin levels in 2 groups treated with intermediate dosages of ruxolitinib (5 mg/kg and 10 mg/kg). The findings suggest that stochastic events such as the wellbeing of the mice or state of haemolysis or difference in drug metabolism might have affected the readings instead of ruxolitinib treatment as the incidence did not correlate with the tested concentrations of the drug. Interestingly, the renal profiles were affected more in the treated groups. The levels of potassium, sodium and phosphate were already increased in the control group and were expectedly influenced by the progesterone and prolactin in the lactating mice that could lead to increased renal reabsorption or reduced excretion^34,35^. Surprisingly, ruxolitinib treatment brought the levels of potassium and phosphorus back or close to normal physiological ranges suggesting a potential role of the drug in regulating renal function that warrant further investigations.

Ruxolitinib targets Jaks particularly with superior potency against the tyrosine kinase domain of Jak2. It has been widely demonstrated to inhibit Jak/Stat signalling pathway or downstream activity but not the level of Jaks expression. To date, no ruxolitinib-Jak co-crystal has been described, and the inhibitory effects were only reported in a handful of human kinases leading to a vast questionable list of off-targets^36^. On the other hand, stabilisation of Jak2 phosphorylation was reported in Burkitt lymphoma cell lines^37^ and human megakaryoblastic cell line (SET-2) treated with ruxolitinib^38^. The resistance to ruxolitinib could occur due to the presence of JAK2 resistance mutation^39^, or the formation of JAK family heterodimers, which means other kinases such as JAK1 and TYK2 might associate with and phosphorylate JAK2 in ruxolitinib treated cells^40^. Besides, ruxolitinib is Type I inhibitors, which binds to kinase active conformation, leading to the stabilisation of activation-loop phosphorylation, thereby resulting in increased phosphorylated levels of JAK2^41^. In this study, we used the drug at the non-effective dosage range during the gestational period to suppress Jak-Stat signalling pathway in the pups' brain. In general, the expression levels of total Jak1 and its corresponding Tyr1034/1035 phosphorylation were not affected by the treatment. However, total Jak2 expression was found in the upward trend in the P1.5 pups' brain correspond to increased concentration of ruxolitinib. The differences became significant at 30 mg/kg/day treatment group, but the corresponding Tyr1007/1008 phosphorylation remained stagnant throughout. We did not screen other phosphorylation sites, and therefore we could not preclude the possibility of phosphorylation at sites other than Tyr1007/1008.

JAK-STAT signalling pathway activation in the brain promotes the production of astrocytes or astrogenesis. Despite the unclear mechanism of ruxolitinib actions in targeting Jak/Stat signalling pathway, the drug has been repeatedly shown effective against myeloproliferative neoplasms and dramatically reduced the associated symptoms in patients^3,42^. When we assessed the treated groups for gliogenesis-suppression, ruxolitinib significantly inhibited the expression of Gfap protein and *S100β* mRNA, and both are markers for astrocytes. A previous study showed downregulation of Jak2 inhibited the transcription of *Gfap*^43^. However, there were no changes to the levels of expression or phosphorylation of Jak2 in our study, suggesting that ruxolitinib did not suppress gliogenesis via inhibition of Jak2 phosphorylation, at least not at Tyr1007/1008. Ruxolitinib has been reported to reduce Gfap expression by 60% in the brain of treated mice^24^. Concurring with the findings, we demonstrated the transplacental suppressive effect of ruxolitinib on astrogenesis during in utero development, an observation, which was validated in differentiating neurospheres cultured ex vivo. More interestingly, ruxolitinib did not affect Tuj1 protein expression but upregulated *Tuj1* (*Tubb3*) and *Neun* (*Rbfox3*) mRNAs, suggesting that it is potentially pro neurogenesis indirectly via the modulation of Jak/Stat-mediated activation of astrogenesis during brain development.

Gliogenesis in mice begins at late embryonic stages (E18 onwards) and peaks during early postnatal development. To our surprise, the suppression of astrogenesis in early development via ruxolitinib treatment led to a lasting effect at least until young adulthood in the context of improved explorative or less anxious behaviour, spatial learning and long-term memory retention. Glial cells play an essential role during embryonic brain development, especially in angiogenesis, axonal outgrowth, synaptogenesis and synaptic pruning^44^. Astrocytes regulate neuroplasticity signalling such as astrocytic Ca^2+^-mediated cholinergic-induced long-term potentiation (LTP) in the cortex and hippocampus^45^, and cannabinoid receptors-mediated hippocampal long-term depression (LTD) and working memory^46^. In this study, we demonstrated ruxolitinib-mediated suppression of astrogenesis in early P1.5 pups' brain, an effect that should have led to reduced learning capability and poorer memory retention in the treated group, but we observed otherwise. We tempted to speculate that the treatment led to collateral benefits for neurones; (1) due to reduced astrocytic pruning, (2) improved nutritional uptake because of the leakier blood–brain barrier and (3) improved neuronal survivability due to reduced astrocytic-activation of microglia-mediated neuroinflammation process. Since ruxolitinib treatment was ceased afterbirth, the astrogenesis was expected to resume, as indicated by the similar levels of Gfap and Tuj1 expression in the adult hippocampus of the control and treated groups. The observation concurs with the reversal of adverse effects after ruxolitinib withdrawal in rats and dogs^23^.

## Conclusions

A strategy to suppress or delay astrogenesis could potentially address the neurogenic-to-gliogenic shifts observed in individuals with Down syndrome^17,20–22^, Noonan syndrome^19^ and disease-related to dysfunctional astrogenesis^10,18^. This study demonstrated the transplacental astrogenesis-suppression effect in the developing embryonic mouse brain and how it could lead to lasting effects into young adulthood after ruxolitinib withdrawal. While transient treatment seems beneficial to various aspects related to improving learning and memory retention, further fundamental investigations on how early suppression could affect the brain postnatally, neuroplasticity throughout maturation and ageing should be evaluated. Nonetheless, the study has opened Pandora's box for ruxolitinib's potential use in neurological disorders, which must cautiously proceed.

## Supplementary Information

Supplementary Information.
