# Supplementary material for: Transient prenatal ruxolitinib treatment suppresses astrogenesis during development and improves learning and memory in adult mice
Source: Sci Rep. 2021 Feb 15;11:3847. doi: 10.1038/s41598-021-83222-z (PMC7884429; doi:10.1038/s41598-021-83222-z)
Supplement: Supplementary file 1 — Supplementary Information. [file 41598_2021_83222_MOESM1_ESM.pdf]

Transient prenatal ruxolitinib treatment suppresses astrogenesis during development and improves learning and memory in adult mice

Han-Chung Lee[1,#], Hamizun Hamzah[1,#], Melody Pui-Yee Leong[1], Hadri Md Yusof[1,2], Omar Habib[1], Shahidee Zainal Abidin[3], Eryse Amira Seth[4], Siong-Meng Lim[5], Sharmili Vidyadaran[6], Mohamad Aris Mohd Moklas[4], Maizaton Atmadini Abdullah[6,7], Norshariza Nordin[1,8], Zurina Hassan[9], Pike-See Cheah[4,8,#,*] and King-Hwa Ling[1,8,#,*]

[1] Department of Biomedical Sciences, Faculty of Medicine and Health Sciences, Universiti Putra Malaysia, 43400 UPM Serdang, Selangor, Malaysia.
[2] Department of Biotechnology, Faculty of Science, Technology, Engineering and Mathematics, International University of Malaya-Wales, 50480, Kuala Lumpur, Malaysia.
[3] Faculty of Science and Marine Environment, Universiti Malaysia Terengganu, 21030 Kuala Nerus, Terengganu, Malaysia.
[4] Department of Human Anatomy, Faculty of Medicine and Health Sciences, Universiti Putra Malaysia, 43400 UPM Serdang, Selangor, Malaysia.
[5] Collaborative Drug Discovery Research, Faculty of Pharmacy, Universiti Teknologi MARA, Cawangan Selangor, Kampus Puncak Alam, 42300 Bandar Puncak Alam, Selangor, Malaysia.
[6] Department of Pathology, Faculty of Medicine and Health Sciences, Universiti Putra Malaysia, 43400 UPM Serdang, Selangor, Malaysia.
[7] Institute of Biosciences, Universiti Putra Malaysia, 43400 UPM Serdang, Selangor, Malaysia.
[8] Genetics and Regenerative Medicine Research Centre, Faculty of Medicine and Health Sciences, Universiti Putra Malaysia, 43400 UPM Serdang, Selangor, Malaysia.
[9] Centre for Drug Research, Universiti Sains Malaysia, 11800 Penang, Malaysia.

[#] These authors contributed equally
* Corresponding authors:
PSC: Email: cheahpikesee@upm.edu.my; Telephone: +603-97692355
KHL: Email: lkh@upm.edu.my; Telephone: +603-97692564

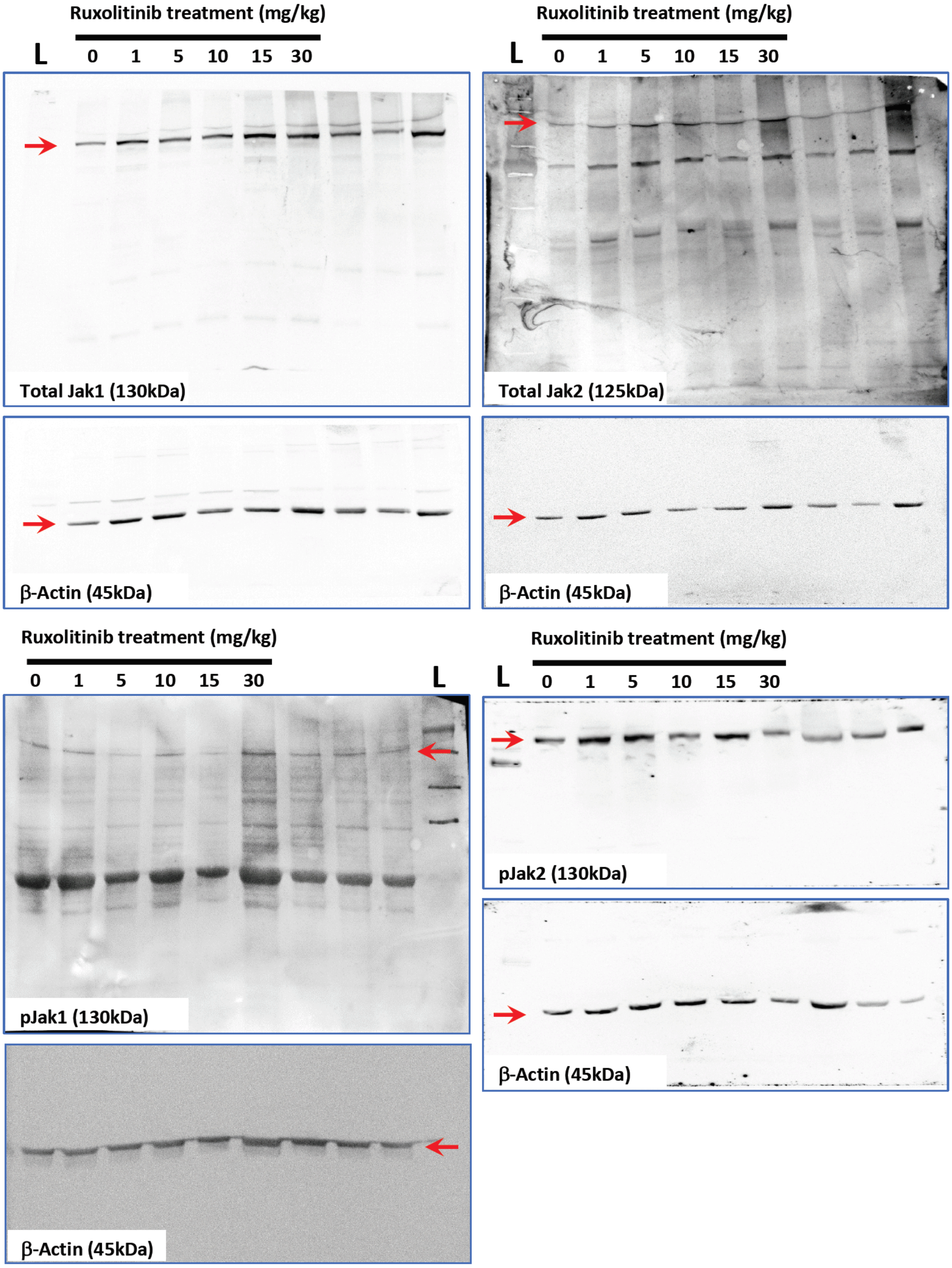

Figure S1: Original immunoblots that correspond to Figure 2C for total Jak1/2 and pJak1/2 in the main manuscript. L denotes the lane whereby the ladder was loaded whereas unlabelled lanes were not presented in the main figure. The red arrows denote the specific bands for the corresponding targets indicated at the lower left of each blot.

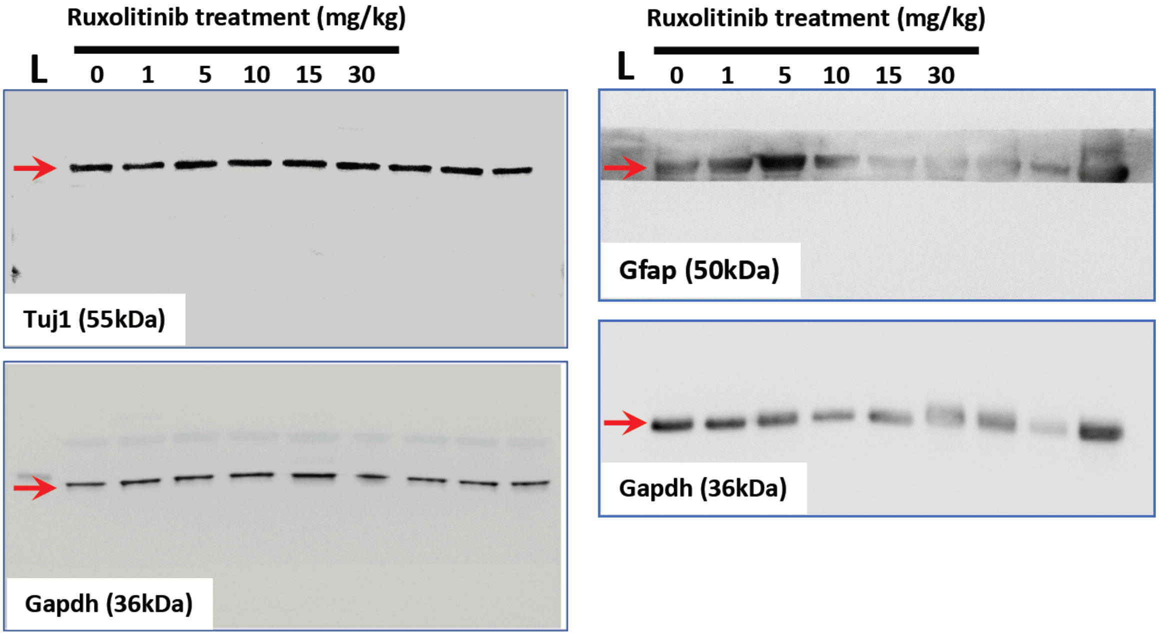

Figure S2: Original immunoblots that correspond to Figure 2D for Tuj1 and Gfap in the main manuscript. L denotes the lane whereby the ladder was loaded whereas unlabelled lanes were not presented in the main figure. The red arrows denote the specific bands for the corresponding targets indicated at the lower left of each blot.

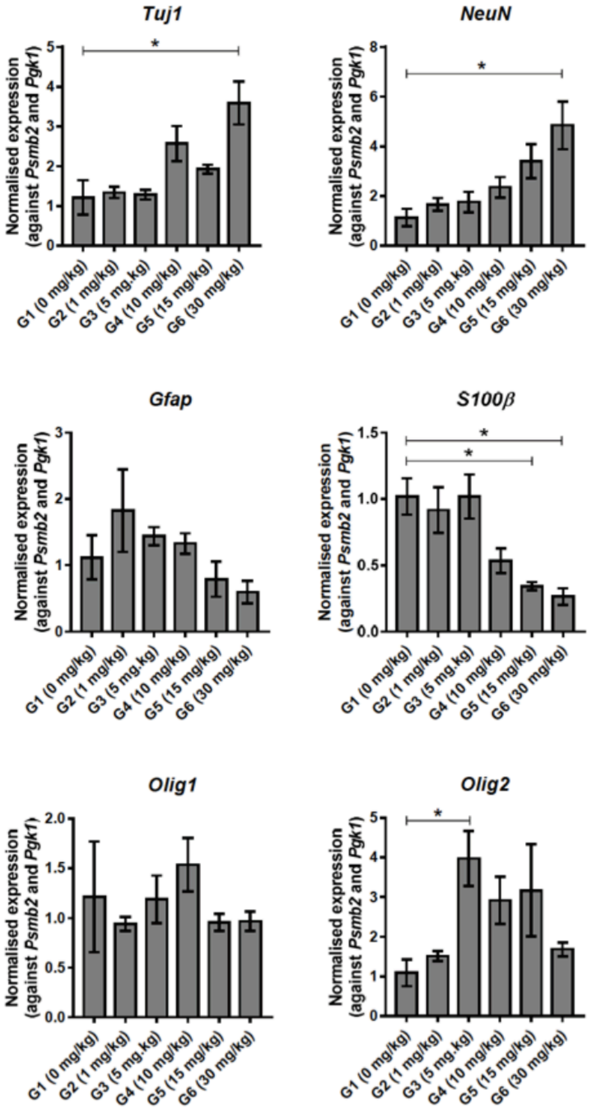

Figure S3: Reverse transcription-quantitative polymerase chain reaction (RT-qPCR) of total RNA isolated from P1.5 whole brain. Gene expression analysis of neuronal cell (*Tuj1* or *Tubb3* and *NeuN* or *Rbfox3*) and glial cell (*Gfap*, *S100β*, *Olig1* and *Olig2*) markers in P1.5 whole brain (n=3 per group) in vehicle control (G1) and all the treated groups (G2-G6). All values represent mean ± SEM; * denotes adjusted $p<0.05$ based on two-tailed, unpaired t-test with Welsh's correction.

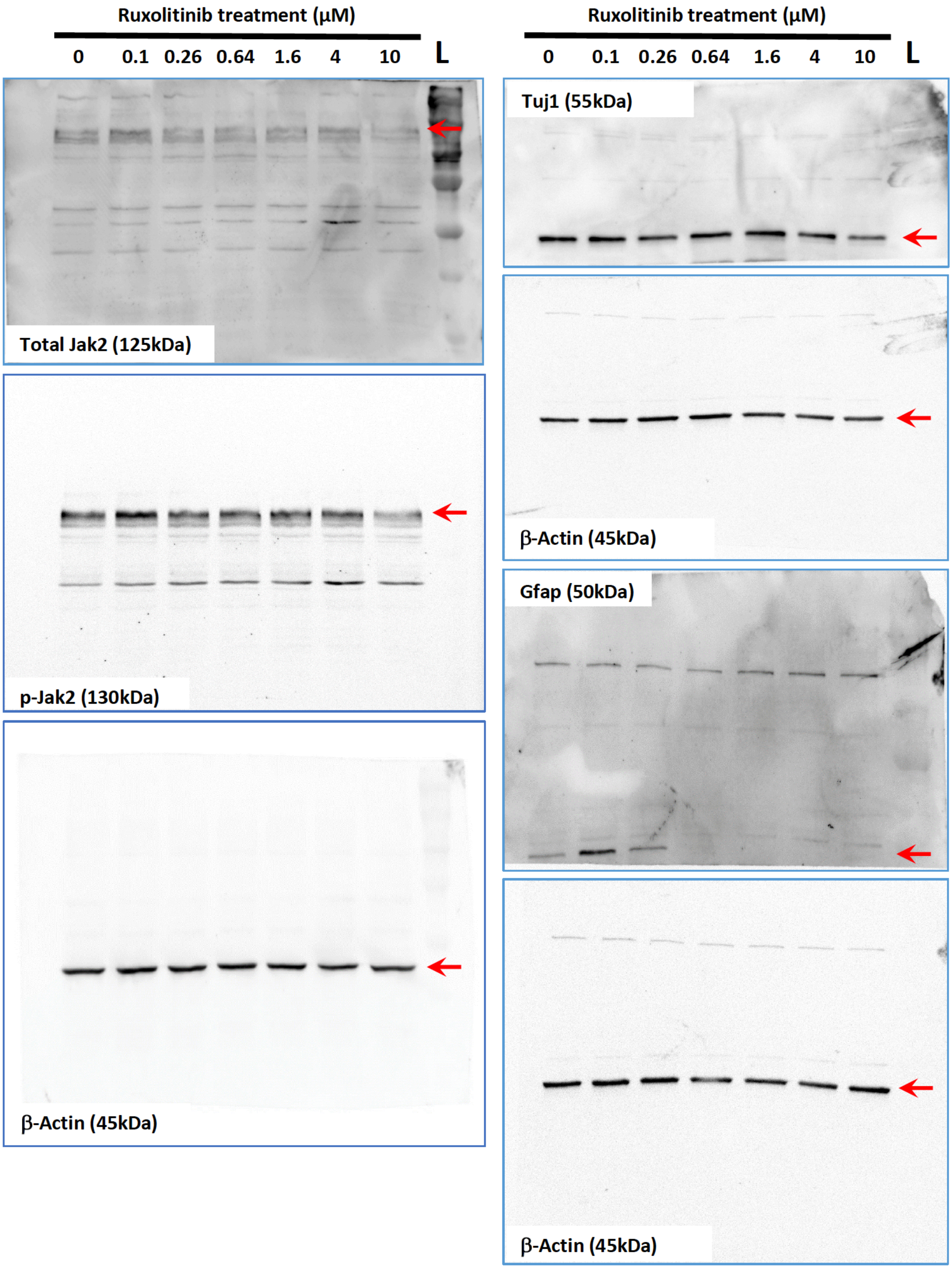

Figure S4: Original immunoblots that correspond to Figure 3B for total Jak2, pJak2, Tuj1 and Gfap in the main manuscript. L denotes the lane whereby the ladder was loaded. The red arrows denote the specific bands for the corresponding targets indicated at the corner of each blot.

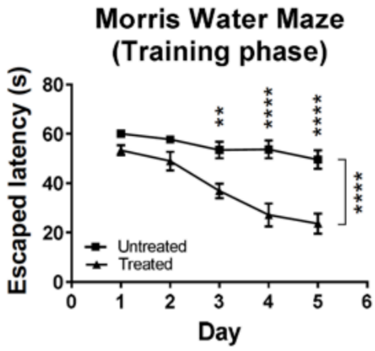

Figure S5: Morris water maze assessment for spatial learning and memory. The escaped latency was measured over a 5-day training course (day 1-5) via multiple entry points. N=14 for the vehicle control group and n=16 for the treated group. All comparisons were tested by using the two-way ANOVA with Sidak's multiple comparisons. All values represent mean±SEM; ** denotes adjusted p<0.01; **** denotes adjusted p<0.0001.
